# Protecting mental health of young adults in COVID-19 pandemic: Roles of different structural and functional social supports

**DOI:** 10.1371/journal.pone.0276042

**Published:** 2022-11-04

**Authors:** Linh Phuong Doan, Long Hoang Nguyen, Ha Ngoc Do, Tham Thi Nguyen, Linh Gia Vu, Huyen Phuc Do, Thuc Minh Thi Vu, Carl A. Latkin, Cyrus S. H. Ho, Roger C. M. Ho

**Affiliations:** 1 Institute for Global Health Innovations, Duy Tan University, Da Nang, Viet Nam; 2 Faculty of Medicine, Duy Tan University, Da Nang, Vietnam; 3 Department of Global Public Health, Karolinska Institutet, Stockholm, Sweden; 4 Youth Research Institute, Ho Chi Minh Communist Youth Union, Hanoi, Vietnam; 5 Vietnam Youth Academy, Hanoi, Vietnam; 6 Center of Excellence in Evidence-based Medicine, Nguyen Tat Thanh University, Ho Chi Minh City, Vietnam; 7 Institute of Health Economics and Technology, Hanoi, Vietnam; 8 Bloomberg School of Public Health, Johns Hopkins University, Baltimore, MD, United States of America; 9 Department of Psychological Medicine, Yong Loo Lin School of Medicine, National University of Singapore, Singapore, Singapore; 10 Institute for Health Innovation and Technology (iHealthtech), National University of Singapore, Singapore, Singapore; University of São Paulo, BRAZIL

## Abstract

**Background:**

Concerning rates of psychological disorders are increasingly recognized in young adults during the COVID-19 pandemic. This study aimed to examine the associations of different structural and functional social supports on depression, anxiety, and stress among young adults in Vietnam.

**Methods:**

An online cross-sectional study was performed on 236 respondents aged 16 to 30 years in Vietnam from June to July 2020. The Depression, Anxiety and Stress Scale—21 Items (DASS-21); the Multidimensional Scale of Perceived Social Support (MSPSS), and the LUBBEN Social Network Scale (LSNS-6) was used to measure psychological health, functional and structural social support characteristics. Multi-level mixed-effect logistic regression was used to identify associations between social support and anxiety, depression, and stress.

**Results:**

The rate of at least mild depression, anxiety, and stress were 30.1%, 34.8%, and 35.6%, respectively. Structural supports measured by LSNS-6 were not associated with the likelihood of having depression, anxiety, and stress (p>0.05). Respondents having friends with whom they could share joys and sorrows were less likely to have anxiety (aOR = 0.61, 95%CI = 0.41–0.90) and stress (aOR = 0.66, 95%CI = 0.45–0.96). Having family support in decision-making was also negatively associated with depression, anxiety, and stress. Meanwhile, those having family and friends who tried to help them were more likely to suffer stress (aOR = 1.94, 95%CI = 1.16–3.24) and depression (aOR = 2.09; 95%CI = 1.11–3.92), respectively.

**Conclusion:**

This study highlighted a high rate of psychological problems among young adults during the COVID-19 pandemic in Vietnam. Emotional support from friends and advice support from family were important components that should be considered in further interventions to mitigate the psychological problems in young adults.

## Introduction

Since the first case was detected in December 2019, the COVID-19 pandemic has devastated lives and economies around the world. It is estimated that as of June 2021, there have been over 175 million cases and 3.8 million deaths due to COVID-19 [1]. Response measures such as social distancing, quarantine, or suspension of economic activities such as tourism and manufacturing widely implemented by governments of different countries [2] alongside the rapid implementation of vaccination campaigns [1] have had substantial effects on pandemic control. However, the emergence of new variants with faster spread rates and greater virulence levels has posed new challenges globally [3, 4], especially for nations with lower COVID-19 vaccination rates such as Southeast Asian and South Asian countries [1]. These variants have been proven to potentially reduce the effectiveness of vaccines [5, 6], which could slow down previous efforts to curb the pandemic. Devastating damages of COVID-19 and uncertainties about its future dynamics have increased the risk of psychological problems in different groups, as well as exacerbated mental disorders in people have who already suffered from psychiatric conditions [7]. Previous reviews have indicated significant increase in the prevalence of mental disorders such as depression (14.6% to 48.3%), anxiety disorders (6.3% to 50.9%), and stress (8.1% to 81.9%) in many countries [7, 8]. Groups with the highest risk of mental disorders are people with chronic illnesses, people in quarantine zones, and COVID-19 patients [8–10].

Youths and young adults are among the most vulnerable individuals during COVID-19 in, as the transition to adulthood presents with unique changes in perceptions and values of life, as well as access to educational and career opportunities [11, 12]. Moreover, common social challenges caused by COVID-19, notably the reduction in job opportunities, income, and social connections, also have profound effects on youths’ psychological well-being. Previous studies have shown that COVID-19 increased the risk of loneliness [13], suicidal ideation [14], unhealthy behaviors and lifestyles [15] as well as worsened sleep quality [16]. Given the severity of such issues, intervention strategies are needed to protect young adults from the devastating impacts of COVID-19 on mental health and to improve their quality of life [17, 18]. Providing appropriate social support is important in reducing the impacts of COVID-19 on mental health. Social support definitions vary widely, but a central conceptual distinction can be made between structural features of social networks, such as the number and diversity of social roles or frequency of social contact that one experiences (i.e., structural support), and the functions of these networks in providing support to an individual (i.e., functional support). Numerous previous studies have shown social support to be negatively correlated with mental disorders such as depression [19–22]. However, these studies have not indicated whether functional support (e.g., emotional or instrumental support) and structural support (eg number of relationships, relationship type) are related specifically to the mental health of the youth population in Asian populations.

The current Vietnamese population structure, in which 45% of its population is under the age of 35, coincides with an economic transformation [23]. This dynamic suggests a pivotal role of Vietnamese youth in the current development of the country. However, considering the socio-economic transformation, increasing risk factors of psychological burden in this population have been neglected. One study measuring the impacts of COVID-19 on the mental well-being of Vietnamese youths found that the rate of anxiety was 9% and there was no association between parent/peer relationships and risk of mental disorders [21]. Acknowledging this knowledge gap, our study aimed to examine the associations of different structural and functional social supports on the depression, anxiety, and stress of young adults in Vietnam.

## Materials and methods

### Ethical approval

This study was approved by the institutional review board of the Youth Research Institute, Vietnam (02-QD/VNCTN) and performed according to the Helsinki declaration guideline [24].

### Study setting and participants

An online cross-sectional study was applied in several provinces of Vietnam from June to July 2020. The eligibility criteria for participating in this survey were: 1) aged from 16 to 30 years (according to the definitions of youths in the Youth Law of Vietnam [25]), 2) who lived in Vietnam for at least 6 months, and 3) agreed to participate in this study by providing online informed consent. Participants who suffered from serious illnesses or could not answer questions from data collectors were excluded from the recruitment process. We used the snowball sampling technique to recruit participants from all provinces of Vietnam. First, we developed a core group of Youth Union leaders in public institutions, companies, and organizations and invited them to complete the survey. The Vietnam Youth Union is the largest group of young adults in Vietnam with more than 7 million members from 14 to 35 years old. After completing the survey, participants invited peers and people in their networks to complete the online survey.

We applied a formula for a proportion to calculate the sample size of this study. With a confidence level of 0.05, the expected of Vietnamese Youth having depression was 39.8% (according to a previous study using DASS-21 [26]), and the relative error level = 0.15. To compensate for the patients who might not answer the questionnaire completely or might end up refusing to participate, an additional 5% was added to the sample size. After calculating, the minimum sample size of this study was 272 participants. At the end of data collection, a total of 236 youths aged 16–30 living in 32/64 provinces of Vietnam completed the survey, with a response rate was 86.7%.

### Measurement and instrument

We created an online questionnaire on SurveyMonkey’s platform (surveymonkey.com) since this approach was considered a low-cost, time-saving, suitable for the youth, and highly accessible approach to reach the samples nationwide during the COVID-19 pandemic. Each participant spent 15–20 minutes completing the survey. We designed a structured questionnaire consisting of four components: 1) socioeconomic characteristics; 2) Current health status; 3) Depression, Anxiety and Stress Scale—21 Items (DASS-21); 4) Social support (MSPSS) and the LUBBEN Social Network Scale (LSNS-6) to measure social network characteristics. The questionnaire was tested and piloted among fifteen youths to examine its appropriateness regarding language and text. Data from the pilot study were not included in the final dataset. After receiving feedback from pilot participants, we revised and uploaded the final version of the questionnaire to the online survey platform. The data collection stage was performed when we ensured that no technical problems could occur. Before collecting data, electronic informed consent was obtained from participants. For those aged below 18 years, we required agreement and confirmation from their parents/guardians to participate in the study.

#### Socioeconomic characteristics and health status

We asked participants to report their information regarding gender, age, household economic, marital status, educational attainment, province, living location, and living arrangement. Furthermore, to explore the information about current chronic diseases and acute symptoms, two questions were developed: 1) “Have you ever been experienced any acute symptoms during the last 4 weeks?” (Yes/No), and 2) “Have you ever been diagnosed with any chronic diseases during the last three months?” (Yes/No). With these above questions, when participants reported that they had been experienced or diagnosed, we would be asked to self-report these symptoms or diseases. These acute symptoms could be included headache, backache, allergy, constipation, cough/sore throat, sneeze/runny nose, fever, helminth infections, diarrhea, gynecologic diseases, skin diseases, eye diseases, etc., that they experienced in the last four weeks and chronic diseases in the last three months. The chronic diseases could have consisted of hypertension, cardiovascular, diabetes, cancer, asthma, epilepsy/psychiatry, stomach/digestive, chronic obstructive pulmonary disease, etc, Participants were grouped into “Having any health problems” if they reported any chronic diseases or acute symptoms.

#### Depression, Anxiety and Stress Scale—21 items (DASS-21)

In this study, we used the DASS-21 instrument to measure the psychological well-being of respondents. This instrument evaluated the depression, anxiety, and stress conditions via 21 items (7 items per domain) with a score ranging from 0 = Never; 1 = Sometimes; 2 = Often; 3 = Almost always. The Cronbach’s alpha values for depression, anxiety, and stress domains were 0.914; 0.864, and 0.909, respectively. The score of each domain was computed by summing scores of items within each domain and then multiplying by 2, resulting in a total score from 0 to 42 in each domain. Each domain of DASS-21 was divided into five levels [27]: Depression: normal (0–9), mild (10–13), moderate (14–20), severe (21–27) and extremely severe (≥ 28), Anxiety: normal (0–7), mild (8–9), moderate (10–14), severe (15–19), and extremely severe (≥ 20), and Stress: normal (0–14), mild (15–18), moderate (19–25), severe (26–33) and extremely severe (≥ 34)

Respondents with mild conditions or above were classified into the “Having depression/anxiety/stress” group. We choose this cutoff as there may be social desirability bias of under-reporting of psychological distress. This scale has been used and validated in several studies in Vietnam [28, 29]. The Cronbach’s value of this scale and each domain of depression, anxiety, and stress in this study were: 0.964; 0.919; 0.878, and 0.914, respectively.

#### Functional and structural social support

*Multidimensional Scale of Perceived Social Support (MSPSS scale)*: Functional social support was evaluated by using the MSPSS scale. This instrument measured the emotional and instrumental support from three sources, including friends, family, and “special person”. It had 12 questions with a 7-point Likert scale ranging from 1 (very strongly disagree) to 7 (very strongly agree), and a higher score revealed a higher level of social support [30]. The Cronbach’s alpha value in this study was 0.976.

*Lubben Social Network Scale-6 (LSNS-6)* was used to measure structural support. This instrument evaluated the social engagement of respondents with family and friends. There were six items with response options varying from 0 (none) to 5 (nine or more). The total score for each domain was the sum of 3 items in each domain, resulting in the total score of each domain ranging from 0 to 15. The total score of the whole scale ranged from 0 and 30, with a higher score indicating more social engagement. A score of 12 or lower delineates “at-risk” for social isolation [31]. The Cronbach’s alpha value in this study was 0.849.

#### Data analysis

The STATA version 16 was utilized to analyze the collected data. Both descriptive and analytical statistics were used to present statistical results. Wilcoxon rank-sum test and χ^2^ test were utilized to compare differences in psychological problems among different characteristics. A p-value (P) <0.05 was considered statistically significant. A two-level mixed-effect logistic regression was used to determine associations between social support with anxiety, depression, and stress. Individuals were treated as level 1, and provinces were level 2. These models were adjusted for socioeconomic characteristics and health status variables. Adjusted Odds ratio and 95% Confidence interval were presented.

## Results

Among 236 participants, the mean age was 20.5 years (SD = 3.6). There were 37.3% of participants living in provinces with COVID-19 cases during the study period. Most of the respondents were aged 16–20 years old (70.3%), females (71.6%), had above high school education (72.0%), lived in the urban area (51.3%), had a spouse/partner (90.7%), living with family (83.9%) and not having any chronic diseases or acute symptoms (57.2%) **(Table 1)**.

**Table 1:** Demographic characteristics of participants (n = 236).

Characteristics	Living in provinces with COVID-19 case				Total		p-value
	No		Yes				
	n	%	n	%	n	%	
**Total**	148	62.7	88	37.3	236	100.0	
**Age group**							
16–20	90	60.8	76	86.4	166	70.3	<0.01
21–25	29	19.6	8	9.0	37	15.7	
26–30	29	19.6	4	4.6	33	14.0	
**Gender**							
Male	39	26.4	28	31.8	67	28.4	0.37
Female	109	73.6	60	68.2	169	71.6	
**Education**							
High school or below	33	22.3	33	37.5	66	28.0	0.01
Above high school	115	77.7	55	62.5	170	72.0	
**Living location**							
Urban area	55	37.2	66	75.0	121	51.3	<0.01
Rural/remote area	93	62.8	22	25.0	115	48.7	
**Marital status**							
Single	129	87.2	85	96.6	214	90.7	0.02
Live with spouse/partner	19	12.8	3	3.4	22	9.3	
**Living arrangement**							
Family	124	83.8	74	84.1	198	83.9	0.95
Others	24	16.2	14	15.9	38	16.1	
**Having any health problems**							
No	85	57.4	50	56.8	135	57.2	0.93
Yes	63	42.6	38	43.2	101	42.8	

The levels of depression, anxiety, and stress among participants were described in **Table 2**. The rate of at least mild depression, anxiety, and stress were 30.1%, 34.8%, and 35.6%, respectively. The mean score for these aspects were 6.8 (SD = 8.9), 6.7 (SD = 8.0), and 9.3 (SD = 9.5), respectively. No difference was found regarding depression, anxiety, and stress between those living in provinces with and without COVID-19 cases (p>0.05).

**Table 2:** Anxiety, depression, and stress patterns (n = 236).

Characteristics	Living in provinces with COVID-19 case				Total		p-value
	No		Yes				
	n	%	n	%	n	%	
**Depression**							
Normal	108	73.0	57	64.8	165	69.9	0.44
Mild	15	10.1	15	17.1	30	12.7	
Moderate	16	10.8	9	10.2	25	10.6	
Severe	2	1.4	3	3.4	5	2.1	
Extremely severe	7	4.7	4	4.46	11	4.7	
**Anxiety**							
Normal	100	67.6	54	61.4	154	65.2	0.64
Mild	9	6.1	4	4.6	13	5.5	
Moderate	21	14.2	18	20.4	39	16.5	
Severe	10	6.8	5	5.7	15	6.4	
Extremely severe	8	5.4	7	7.9	15	6.4	
**Stress**							
Normal	95	64.2	57	64.8	152	64.4	0.75
Mild	32	21.6	16	18.2	48	20.3	
Moderate	12	8.1	11	12.5	23	9.8	
Severe	6	4.1	2	2.3	8	3.4	
Extremely severe	3	2.0	2	2.3	5	2.1	
	**Mean**	**SD**	**Mean**	**SD**	**Mean**	**SD**	
Depression (0–42)	6.6	9.0	7.2	8.7	6.8	8.9	0.50
Anxiety (0–42)	6.4	7.9	7.2	8.2	6.7	8.0	0.50
Stress (0–42)	9.4	9.6	9.2	9.4	9.3	9.5	0.83

**Table 3** showed the average scores of different items of MPSS scales as well as the scores of two LSNS-6 domains in terms of depression, anxiety, and stress conditions. Regarding MSPSS, the mean score of all items was significantly lower among those with depression and anxiety than those without these conditions (p<0.05). Meanwhile, people with stress only had a significantly lower score in three items “I have friends with whom I can share my joys and sorrows”; “There is a special person in my life who cares about my feelings” and “My family is willing to help me make decisions” compared to those without stress (p<0.05). No difference was observed between those with and without depression, anxiety, and stress regarding the score of the “Engagement with family” domain (p>0.05). Meanwhile, the score of domain “Engagement with friends” among those with these conditions was significantly lower than those without conditions (p<0.05).

**Table 3:** Structural and functional supports regarding depression, anxiety, and stress (n = 236).

Characteristics	Depression			Anxiety			Stress		
	No	Yes	p-value	No	Yes	p-value	No	Yes	p-value
	Mean (SD)	Mean (SD)		Mean (SD)	Mean (SD)		Mean (SD)	Mean (SD)	
Multidimensional Scale of Perceived Social Support (MSPSS)									
There is a special person who is around when I am in need.	5.2 (2.0)	4.7 (2.0)	0.03	5.3 (1.9)	4.7 (2.1)	0.01	5.1 (2.0)	4.9 (2.0)	0.28
There is a special person with whom I can share joys and sorrows.	5.2 (1.9)	4.7 (1.9)	0.02	5.3 (1.8)	4.6 (1.9)	<0.01	5.2 (1.9)	4.9 (1.9)	0.17
My family really tries to help me.	5.6 (1.7)	5.1 (1.7)	<0.01	5.6 (1.8)	5.3 (1.6)	0.04	5.5 (1.8)	5.5 (1.5)	0.64
I get the emotional help & support I need from my family.	5.5 (1.8)	4.8 (1.9)	<0.01	5.5 (1.8)	4.9 (1.9)	0.03	5.4 (1.8)	5.2 (1.9)	0.38
I have a special person who is a real source of comfort to me.	5.5 (1.7)	4.8 (2.0)	0.01	5.5 (1.7)	5.0 (1.9)	0.03	5.4 (1.8)	5.2 (1.8)	0.23
My friends really try to help me.	5.3 (1.7)	4.8 (1.7)	0.01	5.3 (1.7)	4.8 (1.7)	0.03	5.2 (1.7)	5.0 (1.6)	0.26
I can count on my friends when things go wrong.	5.0 (1.7)	4.5 (1.9)	0.03	5.0 (1.7)	4.5 (1.9)	0.04	4.9 (1.7)	4.7 (1.9)	0.32
I can talk about my problems with my family.	5.2 (1.7)	4.6 (1.7)	<0.01	5.2 (1.7)	4.7 (1.7)	0.02	5.1 (1.7)	4.9 (1.7)	0.14
I have friends with whom I can share my joys and sorrows.	5.2 (1.8)	4.2 (2.0)	<0.01	5.3 (1.7)	4.2 (2.0)	<0.01	5.2 (1.8)	4.5 (2.0)	<0.01
There is a special person in my life who cares about my feelings.	5.5 (1.8)	4.5 (1.9)	<0.01	5.5 (1.8)	4.7 (1.9)	<0.01	5.4 (1.8)	4.9 (1.9)	0.02
My family is willing to help me make decisions.	5.4 (1.7)	4.4 (1.9)	<0.01	5.5 (1.7)	4.4 (1.9)	<0.01	5.4 (1.7)	4.6 (1.9)	<0.01
I can talk about my problems with my friends.	5.2 (1.7)	4.4 (1.8)	<0.01	5.2 (1.7)	4.6 (1.9)	0.02	5.1 (1.7)	4.8 (1.8)	0.20
Lubben Social Network Scale-6 (LSNS-6)									
Engagement with family	7.9 (3.7)	7.1 (3.4)	0.12	8.0 (3.7)	7.1 (3.3)	0.08	7.9 (3.7)	7.2 (3.3)	0.19
Engagement with friends	7.9 (3.9)	6.8 (3.3)	0.02	7.9 (3.9)	7.0 (3.3)	0.04	7.9 (3.9)	7.0 (3.3)	0.03

The results of the multi-level regression model are shown in **Table 4**. After adjusting for gender, age, household economic, marital status, educational attainment, province, living location and living arrangement, having any health problems, and provinces with COVID-19 cases, structural supports were not associated with the likelihood of having depression, anxiety, and stress (p>0.05). Respondents reporting friends with whom they could share joys and sorrows were less likely to have anxiety (aOR = 0.61, 95%CI = 0.41–0.90) and stress (aOR = 0.66, 95%CI = 0.45–0.96). Reporting family-supporting in decision-making was also negatively associated with depression, anxiety, and stress. Meanwhile, respondents reporting family and friends who tried to help them were more likely to suffer stress (aOR = 1.94, 95%CI = 1.16–3.24) and depression (aOR = 2.09; 95%CI = 1.11–3.92), respectively.

**Table 4:** Multi-level logistic regression for identifying associations between structural and functional social support with depression, anxiety, and stress among young adults.

Types of support	Depression		Anxiety		Stress	
	aOR	95%CI	aOR	95%CI	aOR	95%CI
Multidimensional Scale of Perceived Social Support (MSPSS)						
There is a special person who is around when I am in need.	1.07	0.75; 1.55	0.91	0.67; 1.25	0.98	0.72; 1.35
There is a special person with whom I can share joys and sorrows.	1.56	0.91; 2.68	1.04	0.69; 1.57	1.16	0.77; 1.75
My family really tries to help me.	1.32	0.84; 2.07	1.56	0.98; 2.51	1.94*	1.16; 3.24
I get the emotional help & support I need from my family.	1.00	0.63; 1.57	1.09	0.68; 1.74	1.03	0.65; 1.65
I have a special person who is a real source of comfort to me.	1.27	0.79; 2.05	1.57	0.96; 2.54	1.35	0.82; 2.21
My friends really try to help me.	2.09*	1.11; 3.92	1.41	0.82; 2.43	1.59	0.91; 2.76
I can count on my friends when things go wrong.	1.33	0.83; 2.13	1.08	0.70; 1.68	1.06	0.70; 1.61
I can talk about my problems with my family.	0.71	0.42; 1.21	0.96	0.58; 1.60	0.84	0.50; 1.39
I have friends with whom I can share my joys and sorrows.	0.70	0.48; 1.02	0.61*	0.41; 0.90	0.66*	0.45; 0.96
There is a special person in my life who cares about my feelings.	0.65	0.39; 1.06	0.82	0.52; 1.29	0.84	0.52; 1.35
My family is willing to help me make decisions.	0.49*	0.30; 0.79	0.49*	0.31; 0.77	0.44*	0.27; 0.71
I can talk about my problems with my friends.	0.64	0.41; 1.02	0.89	0.57; 1.39	0.89	0.58; 1.37
Lubben Social Network Scale-6 (LSNS-6)						
Engagement with family	1.09	0.93; 1.27	1.05	0.90; 1.23	1.11	0.95; 1.29
Engagement with friends	0.90	0.77; 1.05	0.93	0.8; 1.08	0.88	0.76; 1.02

* p<0.05; Models were adjusted for gender, age, household economic, marital status, educational attainment, province, living location and living arrangement, having any health problems, and provinces with COVID-19 cases.

aOR: adjusted Odds Ratio

## Discussion

Our study indicated high rates of anxiety, depression, and stress symptoms among youths and young adults in Vietnam regardless of the residential location. Our results also indicated associations between different types of functional social support and mental problems, suggesting further implications to prevent as well as mitigate the consequences of psychological issues.

Results showed that more than a third of young adults reported symptoms of mental health problems such as depression, anxiety, and stress. This result was in line with the rates of mental disorders reported in other studies during the COVID-19 [7, 8]. However, it was significantly higher than previous studies on Vietnam pre-pandemic, which reported the anxiety rate in young people (aged 18 to 26 years) to be 9% [21]. The average scores of these three domains were also higher than that of a previous study on 120 Vietnamese youths (mean stress = 3.8 (SD = 0.53), mean anxiety = 2.10 (SD = 0.44) and mean depression = 2.28 (SD = 0.49) [32]. The discrepancy between studies might lie in the difference between phases of the COVID-19 pandemic in Vietnam. This study was conducted in the early stages of the second wave of COVID-19 in Vietnam, signified by uncontrollable surges in new cases and deaths, while other studies were conducted when the epidemic was or had become stable and under control. Nevertheless, the rate of mental problems in our study was still relatively high compared to studies conducted in the same period. In addition, when comparing rates of mental problems among people living in provinces with and without COVID-19 cases, we observed that rates of depression and anxiety were relatively higher among participants living in provinces with COVID-19 outbreaks than those not living in provinces with pandemic (depression: 35.2% vs. 27%; anxiety: 38.6% vs. 32.4%, respectively). This insignificant difference in data could be attributed to the fact that COVID-19 did not only affect people living in endemic areas but also influenced people living in non-endemic areas with feelings of uncertainty and fear of infections [7, 10].

This study supplemented previous findings on the role of family and friend support. These supports were believed to have positive impacts on the psychological well-being of young people since family members and friends could help young people address feelings of anxiety, depression, or stress [19–22]. However, in this study, the results of the regression model indicated that different forms of support from family and friends had different effects on mental disorders in young adults. We found that during the COVID-19 pandemic, functional supports like “Family really tries to help” or “Friends really try to help” were associated with a higher likelihood of stress and depression in our sample. This finding could be explained in part by the fact that although social support had great advantages in relieving stress, these supports can inadvertently become stressors when young adults feel pressured to meet the expectations of others [18, 33]. This pattern is highly likely to occur in the context of COVID-19 when the impacts of the pandemic and the response measures have taken away young adults’ educational and career opportunities, which were considered by many to be their values in society [11, 12]. Further longitudinal studies should be performed to explore these relationships.

Emotional support from friends, such as sharing joys and sorrows, as well as advisory support from family members in decision making, also played an important role in mitigating the likelihood of psychological disorders in young people. In other words, these supports became stress-buffering factors in helping young adults to be more optimistic and confident in decision-making, and eventually, improve their mental health [18, 34, 35]. The research also showed that structural supports (eg, number of relationships, or frequency of contacts) were not associated with mental health conditions, which might be since COVID-19 has impeded the provision of support and frequency of interactions. Therefore, it is important to develop quality rather than quantity of relationships, meaning youths should foster closer, deeper relationships instead of numerous shallow relationships as a coping mechanism to mental health issues during the COVID-19 pandemic.

Limitations of our study should be noted. As we used the cross-sectional study design, the causal associations between social support and psychological health might not be established. Recall bias could also be possible due to the self-report data collection method. Moreover, other characteristics such as living environment, types of jobs and occupations, habits of using the internet, and digital devices were not fully examined, which might be potential confounding factors. Thus, future research should include these variables to adjust the associations between social support and psychological health in young adults.

## Conclusion

This study highlighted a high rate of psychological problems among young adults during the COVID-19 pandemic in Vietnam. Emotional support from friends and advice support from family was found to be important components. Future interventions should consider these insights to establish effective mitigations to psychological problems in young adults.
